# Tumor size and treatment factors as correlates of 10-year mortality in grade III spinal ependymomas: a nationwide analysis

**DOI:** 10.1038/s41598-025-23070-3

**Published:** 2025-11-10

**Authors:** William ElNemer, Abdul Karim Ghaith, Taha Khalilullah, Lansaol Yang, Khaled Zaitoun, Joseph Dardick, Jawad M. Khalife, A. Karim Ahmed, Yuanxuan Xia, Tej D. Azad, Nicholas Theodore, Daniel Lubelski

**Affiliations:** Department of Neurosurgery, Johns Hopkins University School of Medicine, 600 N. Wolfe Street/Meyer 5-181, Baltimore, MD 21287 USA

**Keywords:** Ependymoma, Spine, Tumor size, Machine learning, Mortality, Survival, Cancer, Oncology

## Abstract

Grade III spinal ependymomas are rare central nervous system tumors with poor prognoses and limited treatment guidance. This study aimed to identify predictors of long-term mortality using a national dataset. The National Cancer Database was queried for patients diagnosed with grade III spinal ependymomas from 2004 to 2017. Demographic, clinical, and treatment variables were analyzed with traditional statistics and machine learning models. Model performance was evaluated with C-index and Integrated Brier Score (IBS), and SHAP analysis was used for feature interpretation. A total of 2,011 patients were included. Surgical resection was performed in 83% of cases, with gross total resection in 96% of those. Radiation was given to 33% (mean 41.4 Gy), and chemotherapy to 18%. Ten-year survival was 75%. Long-term survivors were younger (42 vs. 52 years, *P* < 0.001), had fewer comorbidities (*P* < 0.001), and were more often privately insured. Mortality was higher among those receiving radiation (56% vs. 24%, *P* < 0.001) or chemotherapy (37% vs. 12%, *P* < 0.001). Random Survival Forest was the best-performing model (C-index 0.724; IBS 0.176), identifying tumor size, radiation, chemotherapy, and surgery as key correlates of mortality. Kaplan-Meier analysis showed reduced survival with radiation and chemotherapy, but improved survival with surgery. Stratified analyses revealed diminished surgical benefit in pediatric patients and small tumors. Tumor size emerged as the strongest correlate of mortality. Surgery improves survival, though benefit varies by age and tumor features. Chemotherapy and radiation correlated with higher mortality, underscoring the need for individualized treatment planning and prospective validation.

**Supplementary Information:**

The online version contains supplementary material available at 10.1038/s41598-025-23070-3.

## Introduction

Spinal ependymomas are rare glial tumors that represent a small subset of central nervous system neoplasms in adults. They exhibit considerable clinical and biological heterogeneity, contributing to variability in presentation and outcomes^1–3^. High-grade lesions, particularly WHO grade III ependymomas, are associated with significantly poorer prognoses compared to lower-grade tumors with reported five-year overall survival rates for grade III disease ranging from 46% to 58%^3,4^. Clinical management remains challenging due to the rarity of these tumors, their diverse presentations, and the lack of consensus regarding optimal adjuvant therapy. These challenges are especially pronounced in cases of subtotal resection or tumor recurrence^5,6^.

Multiple population-based and institutional studies have identified key factors that influence survival in patients with spinal ependymoma. Tumor grade, patient age, and extent of surgical resection consistently emerge as independent predictors of mortality, with gross total resection, in particular, conferring a survival advantage across studies^3,7–10^. The benefit of adjuvant radiotherapy is less clear, as it is typically reserved for cases of subtotal resection or high-grade histology^1,4–6,11^. Additionally, socioeconomic factors, such as treatment facility volume and geographic proximity to specialized care, have been shown to impact both treatment decisions and outcomes in this patient population^4^.

Given the rarity of grade III spinal ependymomas and the limitations inherent to single-institution series, large national datasets are critical for meaningful risk stratification and identification of mortality predictors. The National Cancer Database (NCDB) offers a valuable resource for evaluating outcomes and prognostic factors in this population^12,13^. The objective of this study is to investigate variables associated with increased mortality in patients with grade III spinal ependymomas using a robust machine learning approach.

## Methods

### Cohort selection

The National Cancer Database (NCDB) is one of the largest cancer registries in the United States and contains almost 34 million cases from over 1500 hospitals. The data is collected from selected health registries accredited by the American College of Surgeons’ Commission on Cancer (Commission on Cancer | ACS). The NCDB is sponsored by the American College of Surgeons (ACS) and the American Cancer Society (ACS) and gathers data from qualified facilities across the United States.

**Fig. 1:**
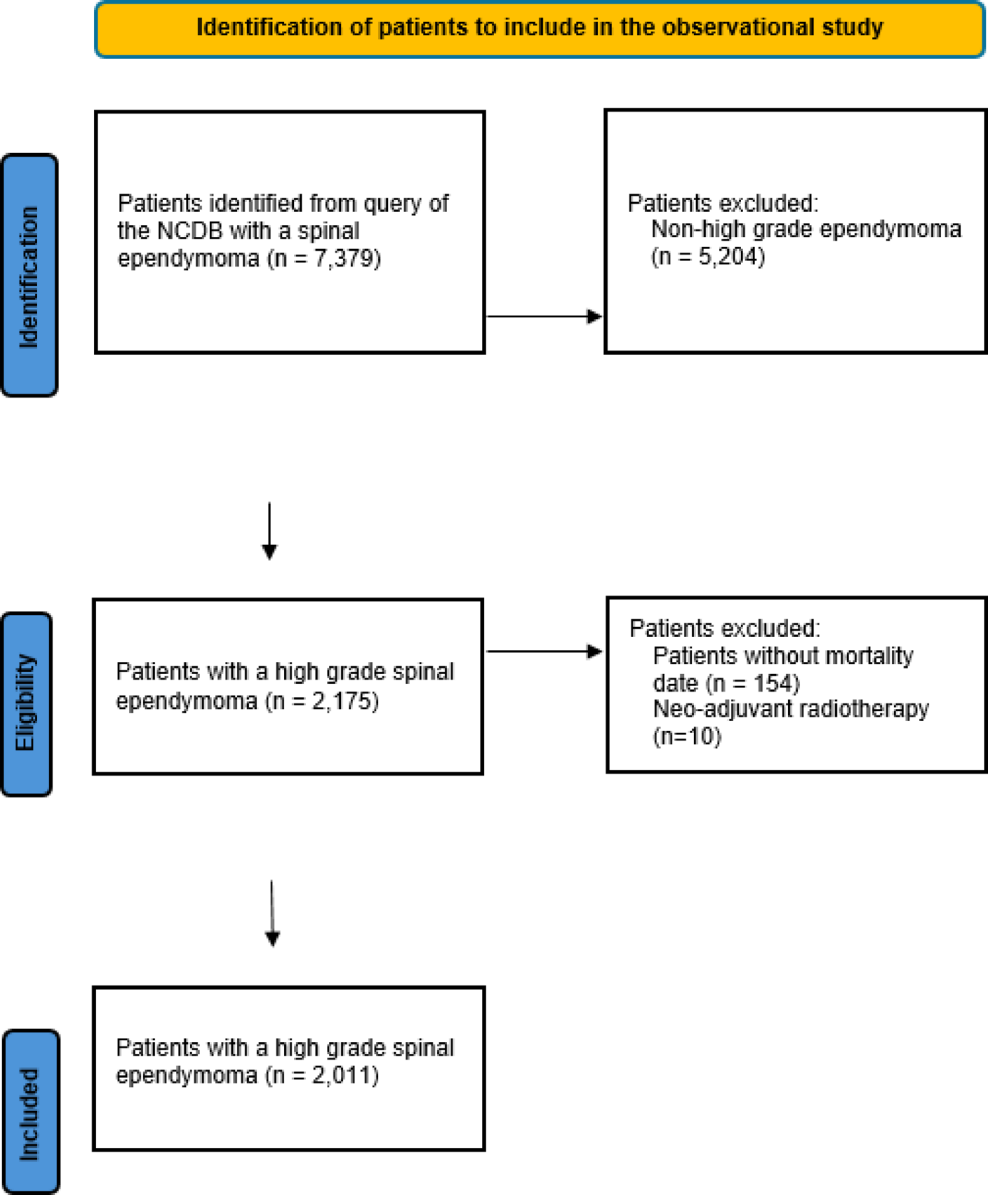
STROBE flowchart for patients included in the observational study.

The NCDB was queried for patients diagnosed and treated for grade III spinal ependymomas from January 1, 2004, through December 31, 2017. We utilized ICD-O-3 topographical codes to identify ependymomas (9383/1, 9391/3, 9392/3, 9393/3, 9394/1, 9396/3) located in the spinal cord (C72.0) and cauda equina (C72.1) by primary site codes. Subsequently, patients with grade I and grade II (low-grade) tumors were excluded in the analysis. Only patients with high-grade, or grade III tumors were included. Patients who received neoadjuvant radiation therapy were excluded due to small sample size and subsequent inability to perform a sub-analysis. A flowchart of inclusion and exclusion criteria is shown in Fig. 1. This study adheres to the Health Insurance Portability and Accountability Act (HIPAA) regulations and aligns with the Strengthening the Reporting of Observational Studies in Epidemiology (STROBE) guidelines. The institutional review board (IRB) classified the study as exempt, as it involved de-identified data and did not require informed consent.

### Patient demographics and disease characteristics

Our study included patient demographics such as age at diagnosis (categorized into adult and pediatric groups), sex (male and female), race (White, Black, Hispanic, and Asian), and insurance status (Private, Medicaid, Medicare, Other Government, and Not Insured). The Charlson-Deyo Comorbidity Classification (CDCC) score was calculated for each patient using the CDCC Mapping Table, categorized as 0, 1, or 2+^14^. Tumor sizes were recorded as the largest diameter of the tumor and they were recorded in millimeters (mm).

### Treatment characteristics

Treatment characteristics included surgical intervention, radiation therapy, and chemotherapy. Radiation was analyzed by technology (photon vs. proton), modality (e.g., brachytherapy, external beam, 3D-conformal), and total dose in a number of fractions. Phase II radiation was defined as a second course after initial therapy. Chemotherapy administration was also recorded.

### Primary and secondary outcome variables

The primary outcome of interest was long-term overall survival (OS) based on the last available clinical encounter. Mortality was defined as death within the latest available follow-up for that patient. Secondary outcomes included 30-day readmission rate, as well as short-term mortality rates documented at 30 days and 90 days. Baseline patient characteristics including demographics, comorbidities, and tumor details were analyzed to evaluate factors influencing the primary outcome.

### Statistical analysis

We performed univariate comparisons of various demographic variables between patients who were alive 10 years after diagnosis, the alive cohort, and those who were not, the mortality cohort. Categorical variables were presented as frequencies and percentages and analyzed using Chi-square tests or Fisher’s exact tests; continuous variables were summarized by means and standard deviations and analyzed using independent samples t-tests for parametric data or Mann-Whitney U tests for nonparametric data. Four machine learning models were employed to predict the OS: the Random Survival Forest model, the Cox Proportional Hazards model, and the Gradient Boosting Survival model. The OS was fit with the following covariates: age (adult vs. pediatrics), number of comorbidities, sex, surgery, chemotherapy, radiation, and tumor size. Missing data can greatly limit machine learning algorithms, so variables such as tumor size which had instances of missing data were imputed with a k-nearest neighbor algorithm^15^.

For the machine learning analysis, we started by partitioning the dataset into training and testing sets, with an 80/20 ratio. To address class imbalance, we applied the Synthetic Minority Over-sampling Technique for Nominal and Continuous (SMOTENC) on the training set to generate synthetic samples for the minority class by creating new instances between existing data points^16^. After data preprocessing, we implemented three primary machine-learning-based survival models: Random Survival Forest, Cox Proportional Hazards, Gradient Boosting Survival, and Random Survival Forest. Model performance was assessed using Receiver Operating Characteristic (ROC) curves. The Concordance index (C-index) evaluates predictive consistency by comparing observed versus predicted survival outcomes in addition to the Integrated Brier Score (IBS). SHapley Additive exPlanations (SHAP) were applied to the best-performing model, as determined by the lowest combination of IBS and C-index, to interpret the influence of each feature on survival predictions.

Finally, the primary outcome (long-term OS) was compared between groups using Kaplan-Meier survival curves and the Log-rank test, with statistical significance defined at *p* < 0.05. In addition, the restricted mean survival time will be calculated for all Kaplan-Meier curves at 100 months as appropriate. All these analyses were repeated for three sub analyses: patients who had surgery, patients who had radiation, and patients who had chemotherapy. These sub analyses were informed by the most important variables as identified by machine learning analysis. In addition, tumor size was analyzed against mortality number and mortality rate in addition to ROC curves to analyze significant cut-offs for tumor size in predicting mortality. All statistical analyses were conducted using RStudio 2025 and Python 3.13.0.

## Results

We identified 2,175 patients with grade III spinal ependymoma, of whom 2,021 had documented mortality status or follow-up dates. An additional 10 patients were excluded due to neoadjuvant therapy (Fig. 1). The mean age was 45 ± 21 years, and 953 (47%) were female. Most patients (*n* = 1,615; 80%) had no comorbidities; 238 (12%) had one, and 158 (8%) had two or more. The mean tumor size was 69 ± 78 mm. Race and insurance distributions are summarized in Table 1.

**Table 1:** Overall characteristics of matched pediatric and adult cohort with grade III spinal Ependymoma.

Characteristics	Total (*N* = 2011)	Alive (*N* = 1504)	Mortality (*N* = 507)	*P*
Age	44.8 ± 20.6	42.4 ± 19.1	51.8 ± 23.1	**< 0.001**
Sex				**0.019**
Female	953 (47%)	736 (49%)	217 (43%)	
Male	1058 (53%)	768 (51%)	290 (57%)	
Race				0.252
Black	204 (10%)	145 (10%)	59 (12%)	
Other	129 (6%)	102 (7%)	27 (5%)	
White	1678 (83%)	1257 (84%)	421 (83%)	
Distance from Hospital (miles)	58 ± 164.7	61.2 ± 171.8	48.8 ± 142.4	0.120
Comorbidity				**< 0.001**
Zero	1615 (80%)	1252 (83%)	363 (72%)	
One	238 (12%)	164 (11%)	74 (15%)	
Two Plus	158 (8%)	88 (6%)	70 (14%)	
Tumor Size (mm)	69.3 ± 78.2	67.9 ± 72.9	95 ± 143.5	0.062
Insurance Status				**< 0.001**
Medicaid	235 (12%)	168 (12%)	67 (14%)	
Medicare	398 (20%)	207 (14%)	191 (39%)	
Not Insured	75 (4%)	59 (4%)	16 (3%)	
Other Government	43 (2%)	33 (2%)	10 (2%)	
Private	1198 (61%)	990 (68%)	208 (42%)	

A total of 1,672 patients (83%) underwent surgical resection, of which 1,604 (96%) were gross total resections (GTR). Chemotherapy was administered to 356 patients (18%), and radiation to 640 (33%). The mean radiation dose was 41.4 ± 14.7 Gy over 22 ± 15 fractions. The distribution of radiation modality and the type of radiation are also shown in Table 1. The average length of stay was 6.6 ± 9.1 days, and 92 (5%) of patients were readmitted within 30 days. In total, 1504 (75%) patients were alive after 10 years while 507 (25%) patients died (Table 2).

**Table 2:** Outcomes of matched pediatric and adult cohort with grade III Ependymoma.

Characteristics	Total (*N* = 2011)	Alive (*N* = 1504)	Mortality (*N* = 507)	*P*
Length Of Stay (days)	6.6 ± 9.1	6.0 ± 7.7	8.8 ± 13	0.185
30-Day Readmission	92 (5%)	66 (4%)	26 (5%)	0.537
30-Day Mortality	15 (1%)	0 (0%)	15 (4%)	**< 0.001**
90-Day Mortality	9 (1%)	0 (0%)	42 (12%)	**< 0.001**
Months Till Death or Last Contact	64 ± 47	76 ± 46	28 ± 28	**< 0.001**

### Comparison of alive and mortality cohort

The average age of the alive cohort was 42 ± 19 years whereas the average age of mortality cohort was 52 ± 23 years (*P* < 0.001). There were 736 (49%) female patients in the alive cohort whereas there were 217 (43%) female patients in the mortality cohort (*P* = 0.019). Patients in the mortality cohort more often had two or greater comorbidities than the alive cohort (14% vs. 6%, *P* < 0.001). In addition, patients in the mortality cohort less often held private insurance (68% vs. 42%, *P* < 0.001) and more often held Medicare (39% vs. 14%, *P* < 0.001) than patients in the alive cohort **(**Table 3**)**.

**Table 3:** Treatment of matched pediatric and adult cohort with grade III Ependymoma.

Characteristics	Total (*N* = 2011)	Alive (*N* = 1504)	Mortality (*N* = 507)	*P*
Surgery	1672 (83%)	1319 (88%)	353 (70%)	**< 0.001**
Surgery Type				0.729
Gross total resection	1604 (96%)	1267 (96%)	337 (95%)	
Subtotal resection	68 (4%)	52 (4%)	16 (5%)	
Chemotherapy	356 (18%)	175 (12%)	181 (37%)	**< 0.001**
Radiation	640 (33%)	356 (24%)	284 (56%)	**< 0.001**
Adjuvant	478 (75%)	286 (78%)	192 (68%)	
Radiation Alone	162 (25%)	70 (22%)	92 (32%)	
Radiation Modality				0.065
3D Conformal Therapy	70 (11%)	44 (12%)	26 (9%)	
External Beam	408 (64%)	218 (60%)	190 (69%)	
Intensity Modulated Therapy	146 (23%)	95 (26%)	51 (19%)	
Stereotactic	11 (2%)	7 (2%)	4 (1%)	
Unknown	5 (1%)	2 (< 1%)	3 (1%)	
Type of Radiation				**0.030**
Photon	561 (97%)	350 (96%)	271 (99%)	
Proton	19 (3%)	16 (4%)	3 (1%)	
Fractions	22.2 ± 15.3	22 ± 17.4	22.4 ± 11.7	0.728
Total Dose (cGy)	4139.6 ± 1467.8	4228 ± 1400.4	4019.6 ± 1549.3	0.084

Patients in the mortality cohort more often received radiation therapy (37% vs. 12%, *P* < 0.001) and chemotherapy (55% vs. 25%, *P* < 0.001) as compared to the alive cohort. In addition, the patients who received radiation therapy in the mortality cohort more often had photon radiation (99% vs. 96%, *P* = 0.030) than patients in the alive cohort **(**Table 3**)**.

### Machine learning analysis.

**Fig. 2:**
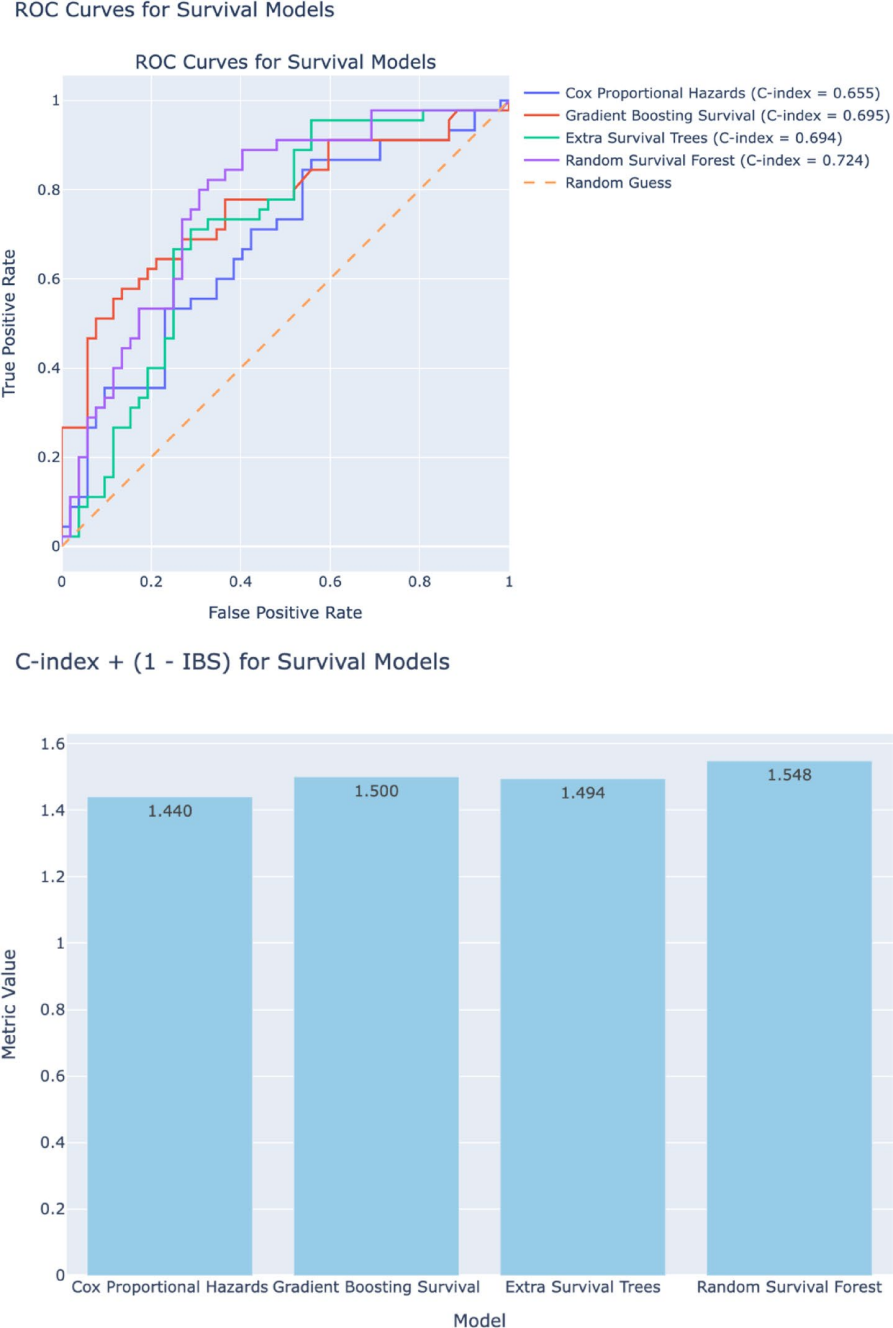
ROC curves and C-index with Integrated Brier Score (IBS) for various machine learning models for survival of patients with grade III spinal ependymoma.

We tested four models and found that Random Survival Forest had the greatest C-index with 0.724, and an IBS of 0.176 (Fig. 1a-b). The larger the C-index the more robust the model is, and the smaller the IBS the more accurate the model, so we used a composite score to choose the model to proceed with. Using this score, the Random Survival Forest model emerged as the best fit model for our data. A Random Survival Forest Model was used with a permutation feature to quantify the decrease in model performance when each variable’s values are randomly permuted, indicating how much the model relies on that feature for accurate survival prediction **(**Fig. 2**)**.

**Fig. 3:**
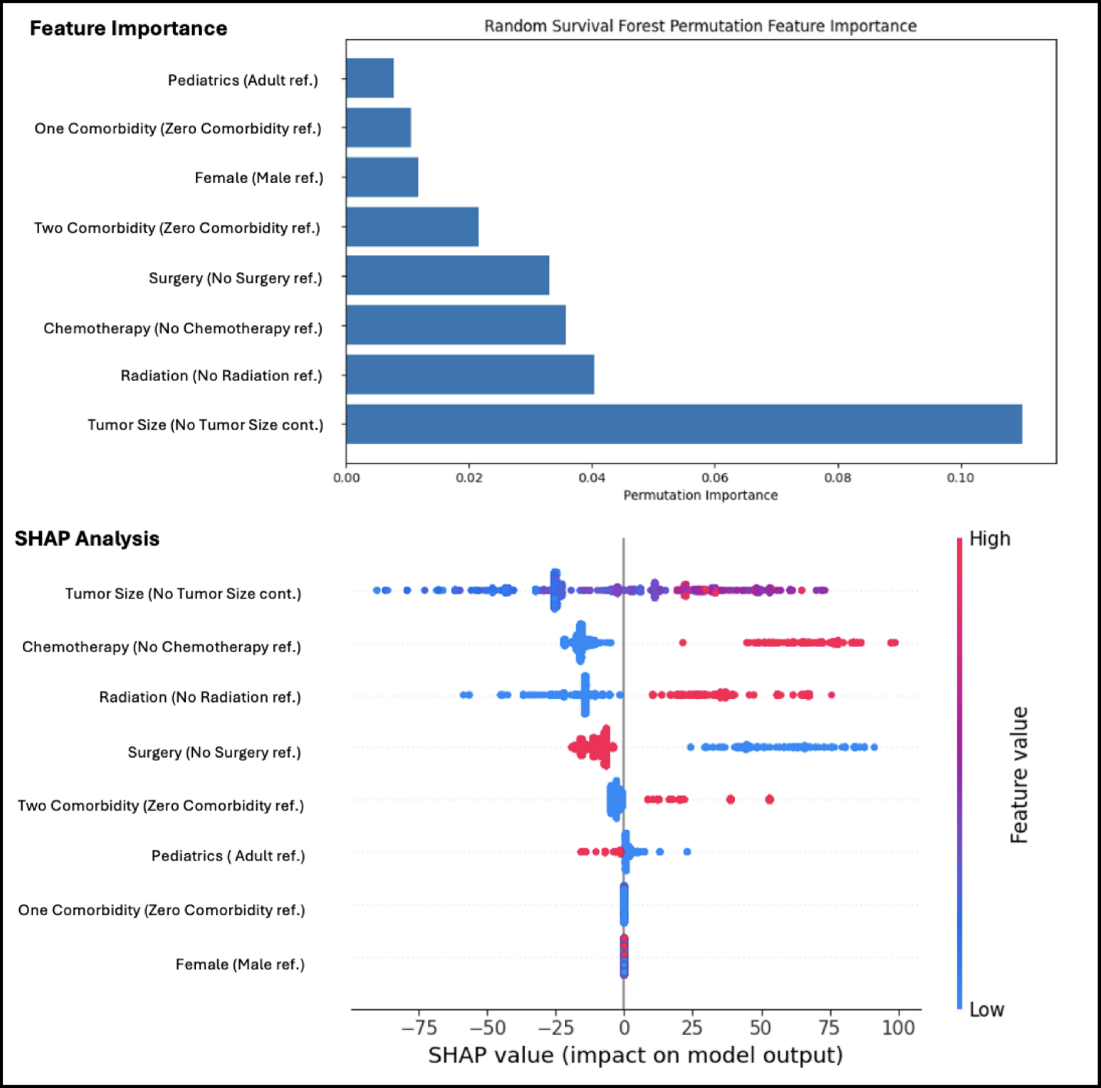
Machine Learning on mortality using random forest survival for whole cohort and summarized with SHAP analysis and feature importance.

In this analysis, among measured variables, tumor size had the highest permutation importance, followed by receipt of radiation, chemotherapy, and surgery. Comorbidity burden, age group, and sex exhibited lower relative contributions to the model’s predictive accuracy. The SHAP summary plot in Fig. 3 shows the distribution and directionality of each feature’s contribution to individual survival predictions. Each point represents a single patient, and its position along the x-axis reflects the magnitude and direction of that feature’s effect on the model output: values to the left correspond to decreased predicted survival, while values to the right correspond to increased predicted survival. The color of each point represents the relative value of the feature, with red indicating high values and blue indicating low values. In general, red values on the right indicate a positive relationship with increased mortality such as increased tumor size, presence of chemotherapy, and presence of radiation therapy. Blue values on the right indicate a negative relationship with increased mortality, thus increasing survival, such as surgery, and, to a lesser degree, pediatric patients.

### Kaplan Meier for whole cohort

**Fig. 4:**
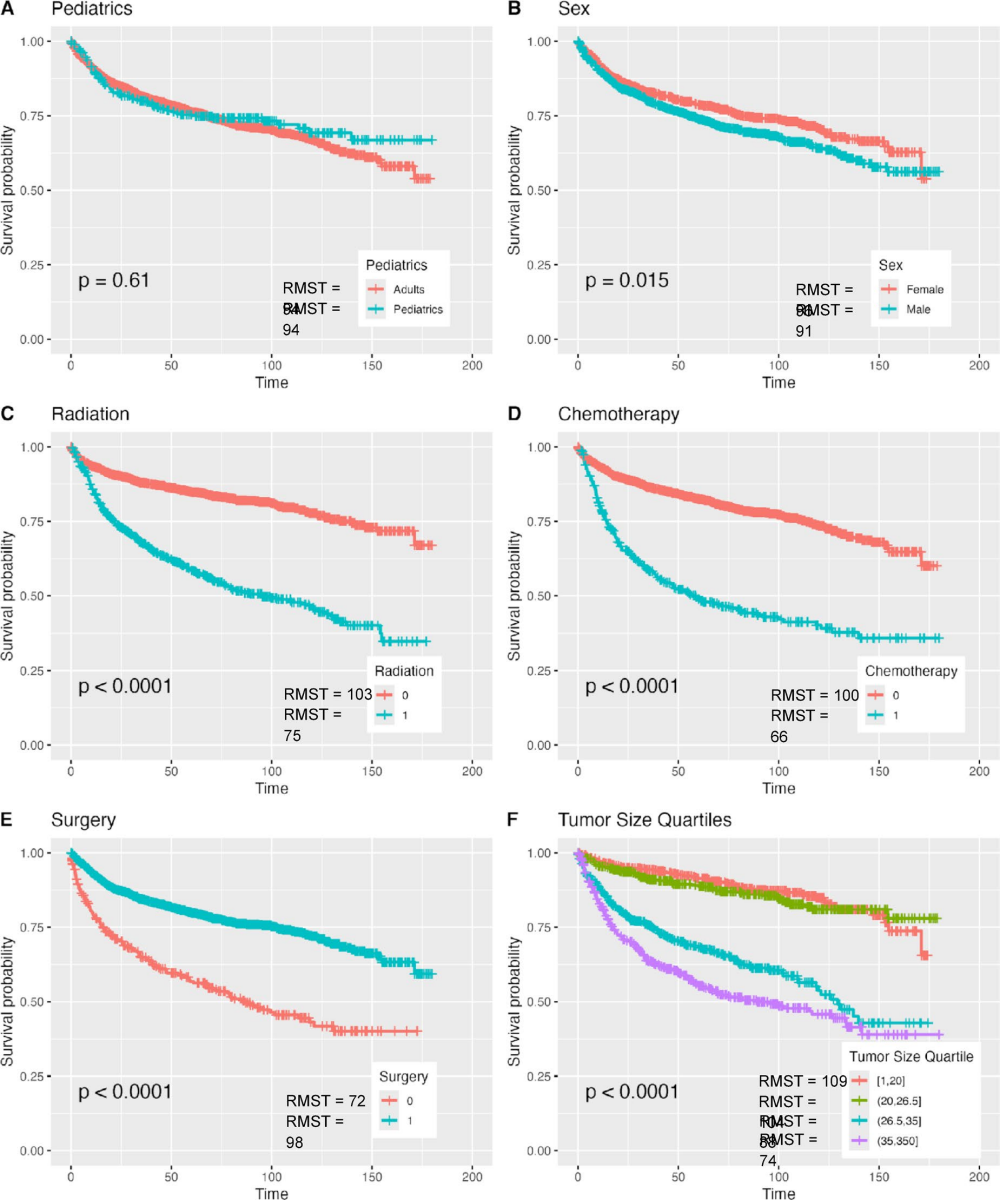
Kaplan Meier Curves for Whole Cohort for Various Patient and Treatment Characteristics.

The 10-year (120 months) RMST was 94 months for both adults and pediatric patients with no significant difference able to be found between each groups’ survival (*P* = 0.610). The 10-year RMST was significantly longer for females than males (96 vs. 91 months, *P* = 0.015). The 10-year RMST in patients who received radiation was significantly shorter than patients who did not (103 vs. 75 months, *P* < 0.001). Patients who received chemotherapy had a significantly shorter 10-year RMST than patients who did not (68 vs. 100 months, *P* < 0.001). The RMST for patients who underwent surgery was significantly longer than in patients who did not (98 vs. 72 months, *P* < 0.001). Finally, in patients with tumor sizes between 1 and 20 mm, and in patients with tumor sizes between 20 and 26.5 mm, the 10-year RMST was 109 and 104, respectively. However, in patients with tumor sizes between 26.5 and 35 mm and in patients with tumor sizes greater than 35 mm, the 10-year RMST was 88 and 74, respectively **(**Fig. 4**)**.

### Stratified Kaplan Meier analysis of whole cohort

**Fig. 5:**
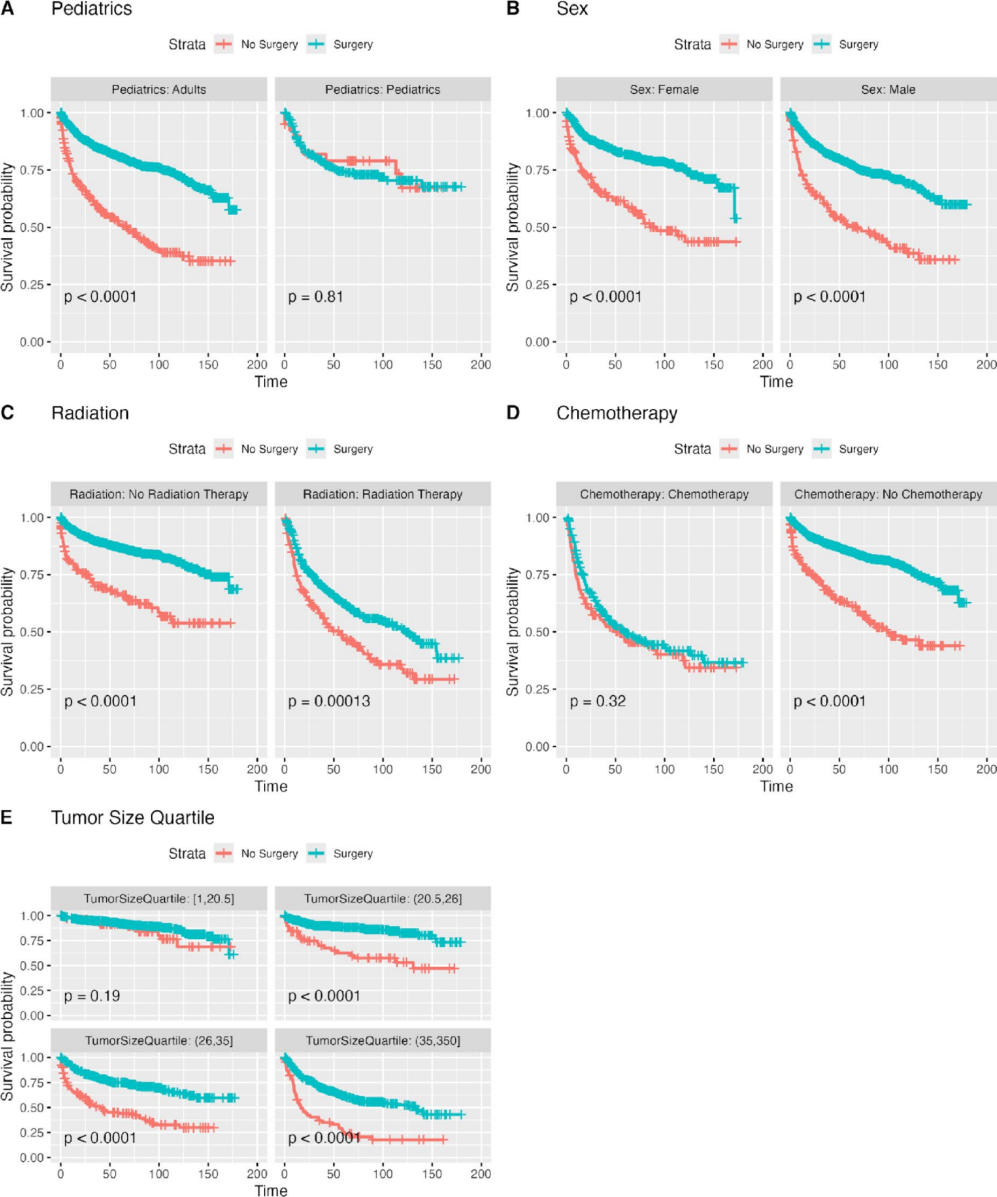
Kaplan Meier Curves for Whole Cohort for Various Patient and Treatment Characteristics Stratified by Surgery or No Surgery.

In pediatric patients who had a larger, but not significantly larger, RMST than pediatric patients who did not undergo surgery (79 vs. 82 months, *P* = 0.810). Notably, surgery was not significantly associated with an increased survival rate in tumors that were in the 1st quartile of tumor sizes between 1 and 20.5 mm (*P* = 0.190) **(**Fig. 5**)**. Further analysis confirmed that radiation therapy and chemotherapy were associated with increased mortality in the pediatric cohort (Supplemental Figs. 5 and 6).

### Kaplan Meier for surgery cohort

Separate analysis was performed for the 1672 patients who underwent resection of their spinal grade III ependymoma. SHAP and feature importance were conducted, preceded by Kaplan Meier analysis. Results were largely unchanged from analysis of the entire cohort **(Supplemental 1 and 2)**. Filtering for patients who underwent surgery allowed comparison of the 1604 (96%) of patients who underwent GTR to the 68 (4%) patients who underwent STR. No significant differences in the analysed variables were found, likely due to the small sample size of patients who underwent STR (Supplemental Fig. 3).

### Kaplan Meier for radiation cohort

**Fig. 6:**
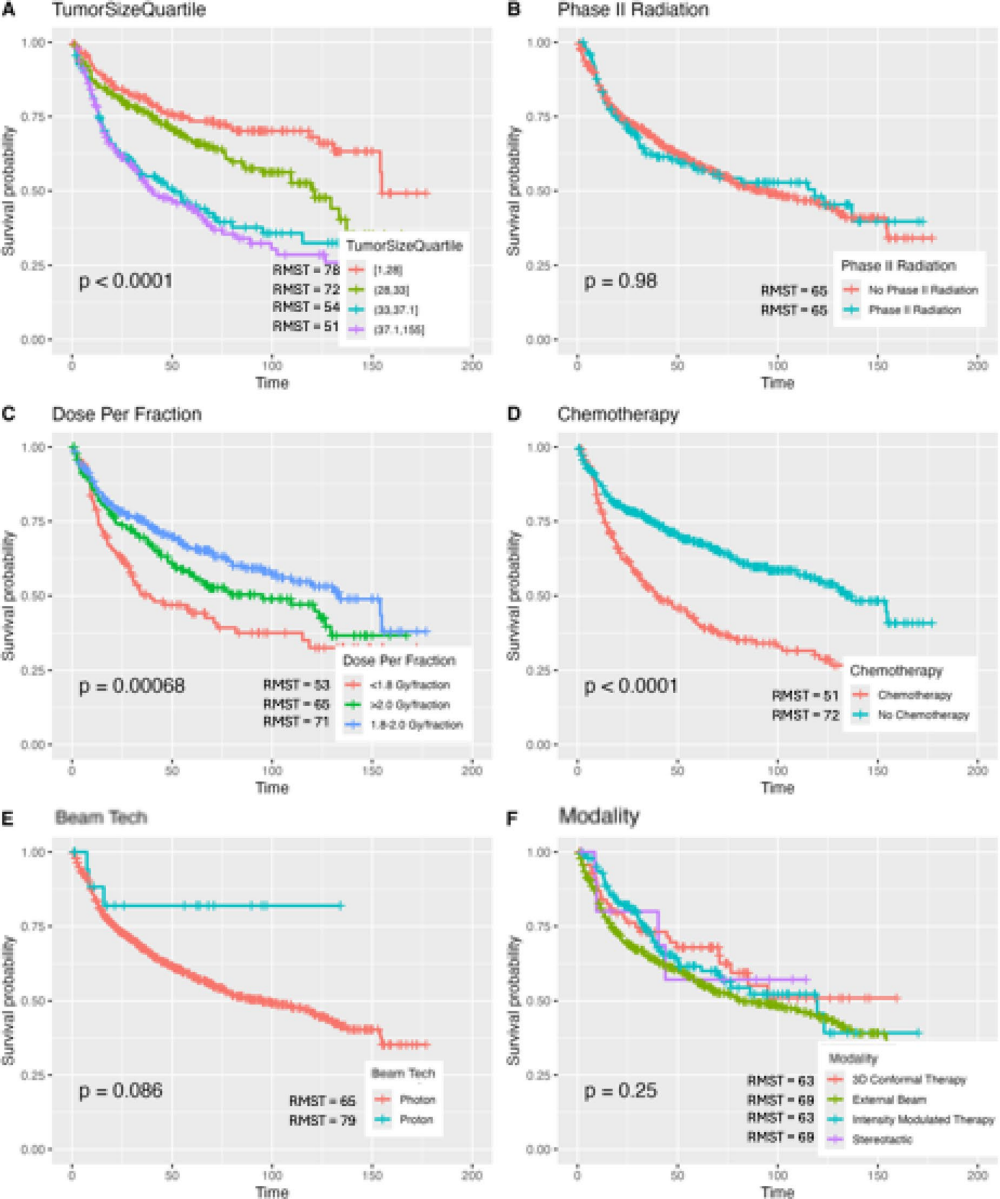
Kaplan Meier Curves for Radiation Cohort for Various Patient and Treatment Characteristics.

The radiation cohort consisted of 640 patients. SHAP and feature importance analysis were conducted (Supplemental Fig. 4). Pediatric patients and sex were not significantly associated with mortality rate in Kaplan Meier analysis. Of note, phase II radiation was not associated with an increased risk of mortality (*P* = 0.980). Interestingly, the standard dose of 1.8–2.0 Gy/fraction was found to have the lowest mortality rate, while dosage at < 1.8 Gy/fraction and > 2.0 Gy/fraction had a greater mortality, with RMSTs of 53, 65, and 71 months, respectively (*P* < 0.001). Beam technology (photon vs. proton) and radiation modality (3-D conformal vs. external beam vs. intensity modulated therapy vs. stereotactic therapy) did not result in significant differences in mortality rate (Fig. 6**)**.

### Kaplan Meier for chemotherapy cohort

**Fig. 7:**
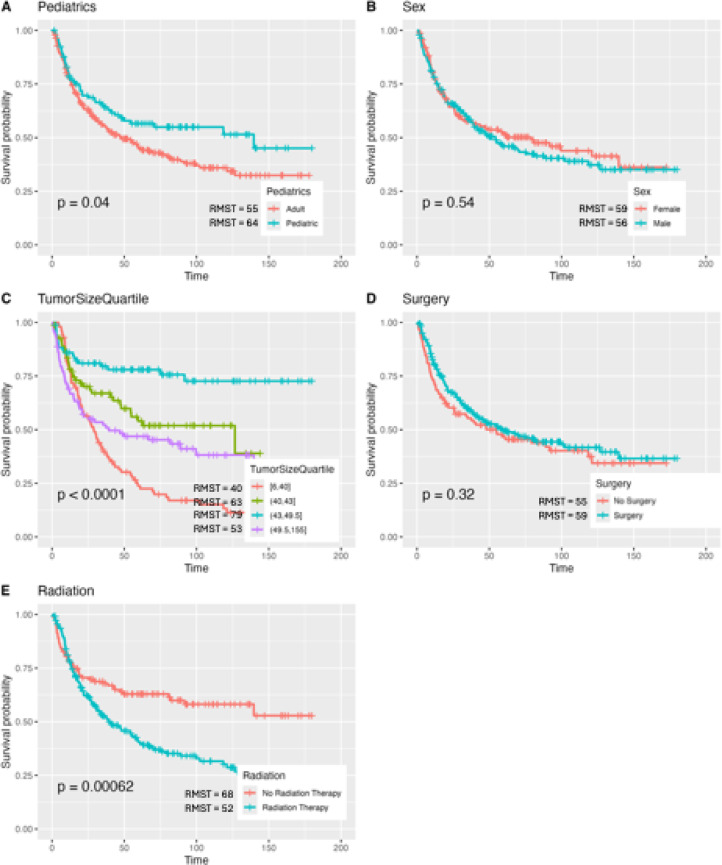
Kaplan Meier curves for chemotherapy cohort for various patient and treatment characteristics.

356 patients in our sample underwent chemotherapy. SHAP and feature importance analysis were conducted with tumor size and presence of radiation measuring as the most important features (Fig. 7). Surgery was not found to be an important feature in this cohort. There were no significant differences detected in mortality rates between males and females or between patients who did or did not undergo surgery (*P* = 0.540 and *P* = 0.320, respectively). However, pediatric patients in this cohort had a significantly longer RMST than adult patients at 64 and 55 months, respectively (*P* = 0.04). Interestingly, the 1st and 4th quartile of tumor size (RMST = 40 and 53 months, respectively) had the greatest mortality risk whereas the 2nd and 3rd quartiles (RMST = 63 and 79 months, respectively) had the least mortality risk (*P* < 0.001).

#### Analysis of tumor size

**Fig. 8:**
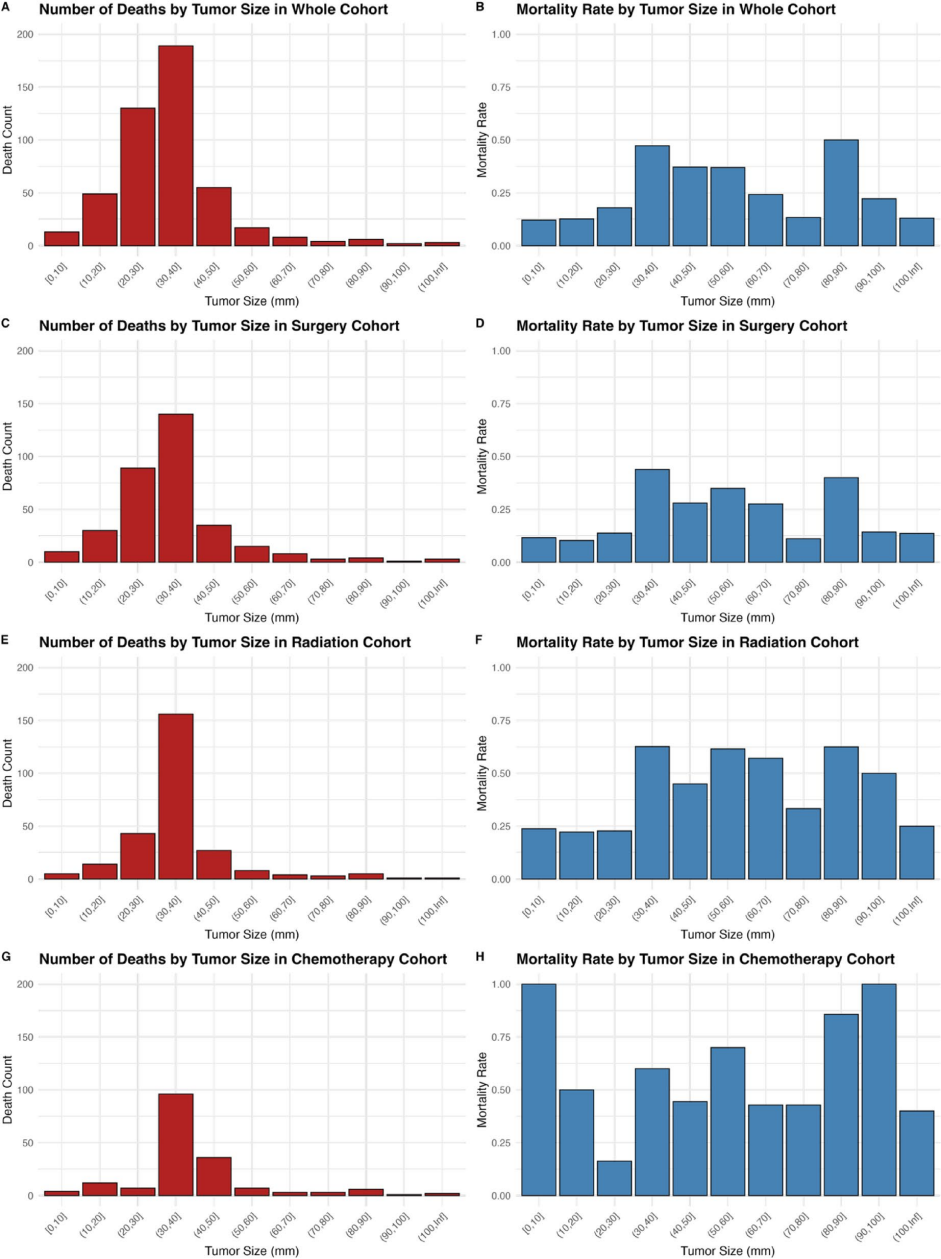
Mortality rate and death number by various cohorts by tumor size.

The number of deaths by 10 mm increments of tumor size are shown in Fig. 8 for the whole surgery, chemotherapy, and radiation cohort in Fig. 8A, C, E, and G. The corresponding mortality rate for each tumor size is shown in Fig. 8B, D, F, and H. Trends largely showed that a larger tumor size was generally associated with an increased mortality rate. For this reason, ROC analysis was conducted for the surgery and radiation cohort to find the optimal cut-off of tumor size that was associated with increased mortality. For the chemotherapy cohort, two ROC analyses were conducted, one for tumor sizes above the median, and one for tumor sizes under the median. In patients undergoing surgery, the ROC analysis revealed a threshold of 24.75 mm, with tumors greater than this volume being associated with a hazard ratio of 5.1 (95% confidence interval (CI): 3.8–6.7) compared to patients with tumors less than 24.75 mm. In patients undergoing radiation therapy, a threshold of 29.75 mm was associated with a hazard ratio of 3.6 (CI: 2.5–5.3) compared with patients with smaller tumors. Finally, in patients undergoing chemotherapy therapy, a threshold of 34.75 mm was associated with a hazard ratio of 3.1 (CI: 2.1–4.6) compared with patients with smaller tumors. Finally, in the chemotherapy cohort, ROC analysis of tumor sizes smaller than the median of 43 mm revealed a threshold of 39.75 mm and analysis of tumor sizes larger than the median revealed a threshold of 51.25 mm. Tumor sizes less than 39.75 mm or greater than 51.25 mm were associated with a 1.9 (CI: 1.3–2.9) increased risk of mortality compared to tumors between 39.75 and 51.25 mm.

## Discussion

Spinal grade III ependymomas represent a rare but clinically aggressive subset of central nervous system tumors, often requiring complex multidisciplinary management. Prior studies have established that tumor grade, surgical resection extent, and select demographic factors influence outcomes, but optimal treatment strategies, particularly regarding adjuvant therapies, remain unclear^1,3,9,10,17^. Given the rarity of these tumors, prior work has been limited by small sample sizes and inconsistent application of advanced analytic methods^5,6,18^. To better inform treatment decisions, we sought to evaluate predictors of long-term survival using a large, nationally representative cohort and modern machine learning techniques to identify clinically relevant patterns. Our analysis revealed that larger tumor size consistently tracked with higher mortality and was among the most influential correlates in the models. In addition, chemotherapy, and radiation were strongly associated with increased mortality, likely due to worse biological factors in the tumors of individuals receiving these therapies, whereas surgical resection, correlated with improved survival. Certain subpopulations like pediatric patients and smaller tumors may not benefit as greatly from resection. In addition, the use of adjuvant radiotherapy must be carefully considered – our analysis revealed that a second phase of radiation was not associated with additional mortality benefit. These findings may go on to inform clinical decision making and be used as a stepping stone for further research.

Surgical resection remains a cornerstone of management in spinal grade III ependymomas, and our findings reaffirm its association with improved long-term survival. This aligns with prior institutional and population-based studies that have demonstrated gross total resection (GTR) as a consistent predictor of favorable outcomes in both cranial and spinal ependymomas^9,10,18–20^. However, our stratified analyses raise important nuances to this recommendation. Among pediatric patients, the survival benefit of surgery was attenuated. This likely multifactorial, driven by both aggressive tumor biology and technical limitations^21–23^. Pediatric tumors often exhibit distinct molecular subtypes associated with higher relapse rates, while anatomical constraints and the risk of neurological morbidity make gross total resection more challenging^24–26^. As a result, even aggressive surgical efforts may yield variable outcomes in this population. Furthermore, the benefit of resection appeared diminished in patients with small tumors, suggesting that tumor volume alone should not automatically trigger operative intervention without careful consideration of neurologic risk and patient-specific context. Smaller tumors may not be as invasive as larger tumors, and may take longer to progress and thus may benefit less from surgical resection, especially in patients who are older and have other comorbidities^6,9,27^. Interestingly, in the chemotherapy subgroup, surgery did not confer a clear survival advantage. This is likely reflective of the disease aggressiveness in these patients. Chemotherapy is not considered a standard primary treatment for grade III spinal ependymomas and is generally reserved for patients with recurrent, refractory, or unresectable disease, or those who are poor surgical candidates due to advanced disease or comorbidities^2,5,6^. As a result, patients who receive chemotherapy typically have more aggressive tumor biology, higher tumor burden, or have failed prior standard therapies, which are all factors associated with worse prognosis and diminished benefit from surgical intervention^2,5,6^. These observations support a more individualized surgical approach, emphasizing the importance of integrating tumor size, age, and planned adjuvant therapies into multidisciplinary treatment planning.

Our analysis found that both adjuvant radiotherapy and chemotherapy were associated with increased mortality, likely reflecting selection bias whereby more aggressive or residual disease prompts escalation of therapy. While radiation in this study was associated with increased mortality, a second phase of radiation was not associated with either increased or decreased risk. This might suggest that even with more severe tumor biology, a second radiation schedule might confer unnecessary morbidity. For example, Brown et al. found no additional survival benefit with adjuvant radiotherapy in surgically resected grade II and III spinal ependymomas, and Keil et al. questioned the utility of postoperative radiotherapy for all cases, even after incomplete resection^28,29^. Similarly, Lee et al. reported that postoperative radiotherapy after incomplete resection did not significantly prolong time to recurrence compared to gross total resection alone^30^. Similarly, various treatment modalities and technologies did not confer worsened or improved mortality in this cohort of patients. This may be limited by sample size of the treatment techniques, specifically proton vs. photon technology. Of note, however, an intermediate dose of 1.8–2.0 Gy/fraction was associated with decreased mortality than smaller or larger doses. While current literature does not compare radiation doses for grade III spinal ependymoma, this phenomenon may be explained by radiobiological principles which suggest that fractionation in this range can maximize tumor control while minimizing normal tissue damage^31,32^. Surprisingly, smaller tumors demonstrated worse outcomes with chemotherapy. This finding may reflect atypical and more aggressive tumor behavior from rapidly progressive molecular subtypes unresponsive to chemotherapy^33^. Much larger tumors are likely more often invasive, and thus chemotherapy may not prevent progression^11,34,35^. These findings highlight the complexity of treatment selection in grade III ependymoma, suggesting that patient and tumor characteristics, rather than specific adjuvant modalities or technologies, may be the primary determinants of mortality.

### Limitations

This study has several limitations inherent to retrospective analyses using national databases. The NCDB lacks granular clinical details such as tumor location, neurologic status, molecular subtype, and recurrence data, which are critical for understanding disease behavior and treatment response. Selection bias is likely, as patients receiving chemotherapy or radiation may have had more aggressive or unresectable disease, confounding associations with mortality. Functional outcomes and progression-free survival could not be assessed. While subtotal resection status was available, the sample size was too small to find any significant differences. Despite the use of advanced machine learning, model performance is limited by the quality and completeness of input data. As such, these findings should be interpreted as associations rather than causative relationships, and future prospective studies with integrated clinical, radiographic, and molecular data are needed to validate these results. The last notable limitation of this study is the relatively small sample size, which may limit the generalizability of the findings. As such, this work should be viewed as an exploratory and hypothesis-generating preliminary study, intended to inform future research more granular data than available in a national database.

## Conclusion

Considering our findings, surgeons managing patients with grade III spinal ependymomas should prioritize individualized treatment planning that considers tumor size, patient age, and comorbidity burden when determining the potential benefit of surgical intervention. Gross total resection remains a cornerstone of care, but our results suggest it may offer limited survival benefit in pediatric patients and those with small or biologically aggressive tumors. We recommend a cautious approach to adjuvant therapies, as chemotherapy and radiation were associated with increased mortality, likely reflecting underlying disease severity, highlighting the need to better define which patients may truly benefit from these modalities. Future research should focus on integrating molecular and genetic profiling to improve risk stratification, understanding the role of tumor location and neurologic function in guiding resection, and prospectively evaluating the impact of dose and modality selection in radiotherapy. Prospective, multi-institutional studies incorporating functional and quality-of-life outcomes are urgently needed to refine therapeutic strategies and optimize long-term care in this vulnerable population.

## Supplementary Information

Below is the link to the electronic supplementary material.

Supplementary Material 1

## Data Availability

The datasets generated during and/or analysed during the current study are available in the NCDB repository, [https://www.facs.org/](https:/www.facs.org) .

## References

[1] Lin, Y. et al. Predictors of survival in patients with spinal ependymoma. *Neurol. Res.* **37**, 650–655 (2015). 25917046 10.1179/1743132815Y.0000000041

[2] Reni, M., Gatta, G., Mazza, E. & Vecht, C. Ependymoma. *Crit. Rev. Oncol. Hematol.* **63**, 81–89 (2007). 17482475 10.1016/j.critrevonc.2007.03.004

[3] Khalid, S. I. et al. Adult spinal ependymomas: an epidemiologic study. *World Neurosurg.* **111**, e53–e61 (2018). 29225135 10.1016/j.wneu.2017.11.165

[4] Yeboa, D. N. et al. National patterns of care in the management of world health organization grade II and III spinal ependymomas. *World Neurosurg.* **124**, e580–e594 (2019). 30641236 10.1016/j.wneu.2018.12.159

[5] Rudà, R. et al. EANO guidelines for the diagnosis and treatment of ependymal tumors. *Neuro Oncol.* **20**, 445–456 (2018). 29194500 10.1093/neuonc/nox166 PMC5909649

[6] Cerretti, G. et al. Spinal ependymoma in adults: from molecular advances to new treatment perspectives. *Front. Oncol.* **13**, 1301179 (2023). 38074692 10.3389/fonc.2023.1301179 PMC10704349

[7] Fu, T. et al. Disease characteristics and clinical specific survival prediction of spinal ependymoma: a genetic and population-based study. *Front. Neurol.* **15**, 1454061 (2024). 39346772 10.3389/fneur.2024.1454061 PMC11428185

[8] Ryu, S. M., Lee, S-H., Kim, E-S. & Eoh, W. Predicting survival of patients with spinal ependymoma using machine learning algorithms with the SEER database. *World Neurosurg.* **124**, e331–e339 (2019). 30597279 10.1016/j.wneu.2018.12.091

[9] Savoor, R. et al. Long-term outcomes of spinal ependymomas: an institutional experience of more than 60 cases. *J. Neurooncol*. **151**, 241–247 (2021). 33179213 10.1007/s11060-020-03658-7

[10] Naito, K. et al. Predictors of progression-free survival in patients with spinal intramedullary ependymoma: A multicenter retrospective study by the neurospinal society of Japan. *Neurosurgery* **93**, 1046–1056 (2023). 37255289 10.1227/neu.0000000000002538

[11] Lopez, C. D. et al. Recent trends in medicare utilization and reimbursement for orthopaedic procedures performed at ambulatory surgery centers. *J. Bone Joint Surg. Am.* **103**, 1383–1391 (2021). 33780398 10.2106/JBJS.20.01105

[12] Lin, C-H-A. & Berger, M. S. Advancing neuro-oncology of glial tumors from big data and multidisciplinary studies. *J. Neurooncol*. **146**, 1–7 (2020). 31853838 10.1007/s11060-019-03369-8

[13] Martin, A. N., Chan, N. W., Cheung, D. C. & Fong, Z. V. A guide to large data sets for population-based cancer research: Strengths, limitations, and pitfalls. *Cancer* **130**, 3802–3814 (2024). 39158578 10.1002/cncr.35535

[14] Deyo, R. A., Cherkin, D. C. & Ciol, M. A. Adapting a clinical comorbidity index for use with ICD-9-CM administrative databases. *J. Clin. Epidemiol.* **45**, 613–619 (1992). 1607900 10.1016/0895-4356(92)90133-8

[15] Tavazzi, E. et al. Exploiting mutual information for the imputation of static and dynamic mixed-type clinical data with an adaptive k-nearest neighbours approach. *BMC Med. Inf. Decis. Mak.* **20**, 174 (2020). 10.1186/s12911-020-01166-2 PMC7439551 32819346

[16] Koivu, A., Sairanen, M., Airola, A. & Pahikkala, T. Synthetic minority oversampling of vital statistics data with generative adversarial networks. *J. Am. Med. Inf. Assoc.* **27**, 1667–1674 (2020). 10.1093/jamia/ocaa127 PMC7750982 32885818

[17] Chen, P. et al. An integrative analysis of treatment, outcomes and prognostic factors for primary spinal anaplastic ependymomas. *J. Clin. Neurosci.* **22**, 976–980 (2015). 25769252 10.1016/j.jocn.2014.11.032

[18] Davison, M. A. et al. Clinical presentation and extent of resection impacts progression-free survival in spinal ependymomas. *J. Neurooncol*. **167**, 437–446 (2024). 38438766 10.1007/s11060-024-04623-4 PMC11096218

[19] Narin, F. et al. Evaluation of pediatric spinal ependymomas: A 25-year retrospective observational study. *Med. (Baltim).* **103**, e40986 (2024). 10.1097/MD.0000000000040986 PMC11666201 39705486

[20] Tarapore, P. E. et al. Pathology of spinal ependymomas: an institutional experience over 25 years in 134 patients. *Neurosurgery* **73**, 247–255 (2013). discussion 255. 23670032 10.1227/01.neu.0000430764.02973.78

[21] Cohen, A. R. Brain tumors in children. *N Engl. J. Med.* **386**, 1922–1931 (2022). 35584157 10.1056/NEJMra2116344

[22] Lampros, M., Vlachos, N. & Alexiou, G. A. Ependymomas in children and adults. *Adv. Exp. Med. Biol.* **1405**, 99–116 (2023). 37452936 10.1007/978-3-031-23705-8_4

[23] Jünger, S. T., Timmermann, B. & Pietsch, T. Pediatric ependymoma: an overview of a complex disease. *Childs Nerv. Syst.* **37**, 2451–2463 (2021). 34008056 10.1007/s00381-021-05207-7 PMC8342354

[24] Safaee, M. et al. Surgical outcomes in spinal cord ependymomas and the importance of extent of resection in children and young adults. *J. Neurosurg. Pediatr.* **13**, 393–399 (2014). 24506340 10.3171/2013.12.PEDS13383

[25] Szathmari, A. et al. Ependymoma of the spinal cord in children: A retrospective French study. *World Neurosurg.* **126**, e1035–e1041 (2019). 30877001 10.1016/j.wneu.2019.03.033

[26] Safaee, M. et al. Histologic grade and extent of resection are associated with survival in pediatric spinal cord ependymomas. *Childs Nerv. Syst.* **29**, 2057–2064 (2013). 23677177 10.1007/s00381-013-2149-x

[27] Wild, F. et al. Surgical treatment of spinal ependymomas: experience in 49 patients. *World Neurosurg.* **111**, e703–e709 (2018). 29309981 10.1016/j.wneu.2017.12.159

[28] Brown, D. A. et al. Radiotherapy in addition to surgical resection May not improve overall survival in WHO grade II spinal ependymomas. *Clin. Neurol. Neurosurg.* **189**, 105632 (2020). 31862631 10.1016/j.clineuro.2019.105632

[29] Keil, V. C. et al. Optimising treatment strategies in spinal ependymoma based on 20years of experience at a single centre. *J. Clin. Neurosci.* **29**, 52–58 (2016). 26944215 10.1016/j.jocn.2016.01.003

[30] Lee, S-H. et al. Long-term outcomes of surgical resection with or without adjuvant radiation therapy for treatment of spinal ependymoma: a retrospective multicenter study by the Korea spinal oncology research group. *Neuro Oncol.* **15**, 921–929 (2013). 23576600 10.1093/neuonc/not038 PMC3688015

[31] Little, M. P., Hamada, N. & Cullings, H. M. Analysis of departures from linearity in the dose response for Japanese atomic bomb survivor solid cancer mortality and cancer incidence data and assessment of low-dose extrapolation factors. *Radiat. Res.* **203**, 115–127 (2025). 39799958 10.1667/RADE-24-00202.1

[32] Haley, B. et al. Findings from international archived data: fractionation reduces mortality risk of ionizing radiation for total doses below 4 Gray in rodents. *Mutat. Res. Genet. Toxicol. Environ. Mutagen.* **882**, 503537 (2022). 36155139 10.1016/j.mrgentox.2022.503537

[33] Pohl, L. C. et al. Molecular characteristics and improved survival prediction in a cohort of 2023 ependymomas. *Acta Neuropathol.* **147**, 24 (2024). 38265522 10.1007/s00401-023-02674-x PMC10808151

[34] Zhao, F. et al. Survival and prognostic factors of adult intracranial ependymoma: A single-institutional analysis of 236 patients. *Am. J. Surg. Pathol.* **45**, 979–987 (2021). 33739788 10.1097/PAS.0000000000001669

[35] Hollon, T. et al. Supratentorial hemispheric ependymomas: an analysis of 109 adults for survival and prognostic factors. *J. Neurosurg.* **125**, 410–418 (2016). 26745489 10.3171/2015.7.JNS151187

