# Supplementary material for: Tumor size and treatment factors as correlates of 10-year mortality in grade III spinal ependymomas: a nationwide analysis
Source: Sci Rep. 2025 Nov 10;15:39318. doi: 10.1038/s41598-025-23070-3 (PMC12603031; doi:10.1038/s41598-025-23070-3)

Supplemental 1: Machine Learning on Mortality using Random Forest Survival for Surgery Cohort and Summarized with SHAP Analysis and Feature Importance.


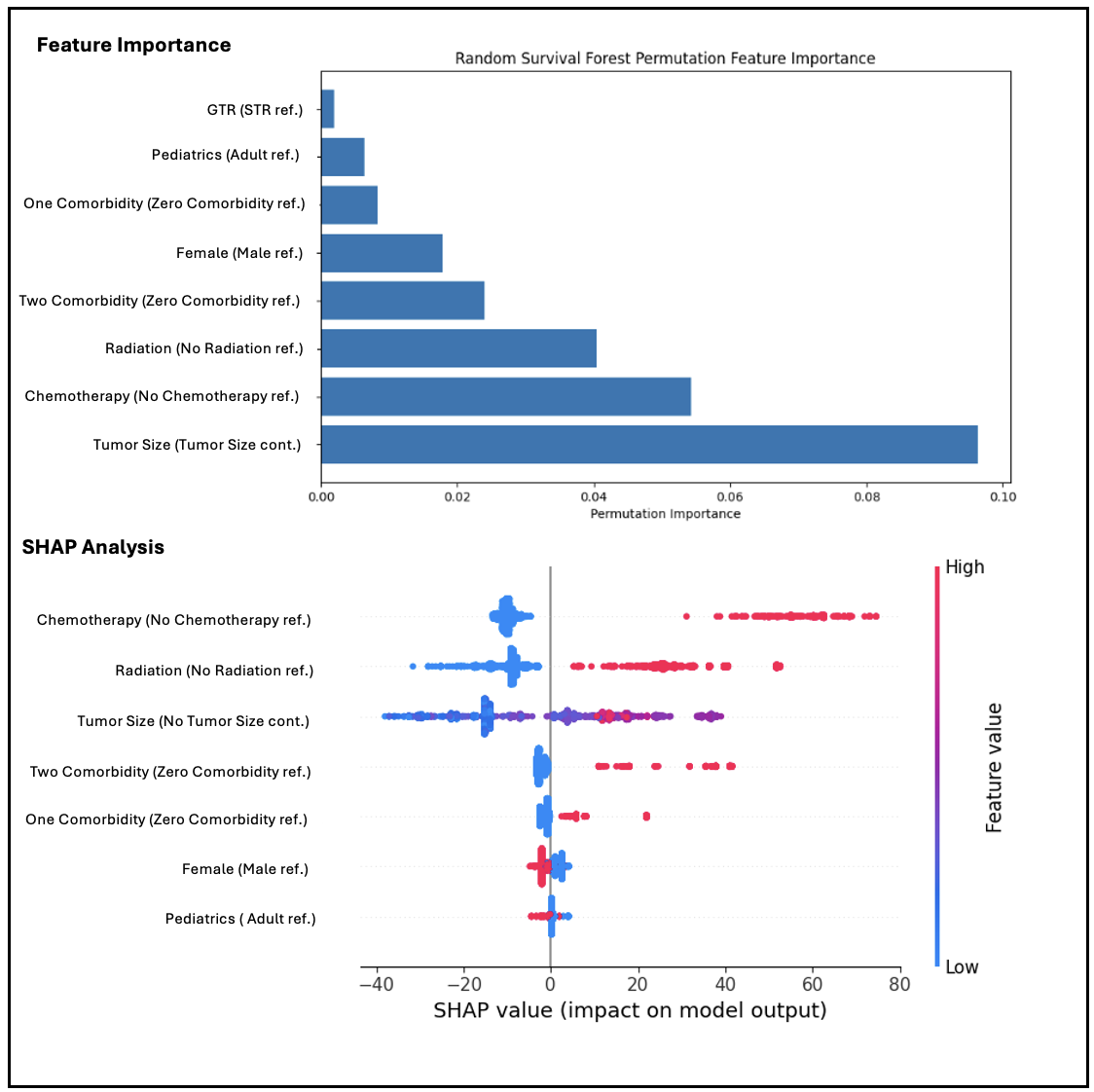


Supplemental 2: Kaplan Meier Curves for Surgery Cohort for Various Patient and Treatment Characteristics


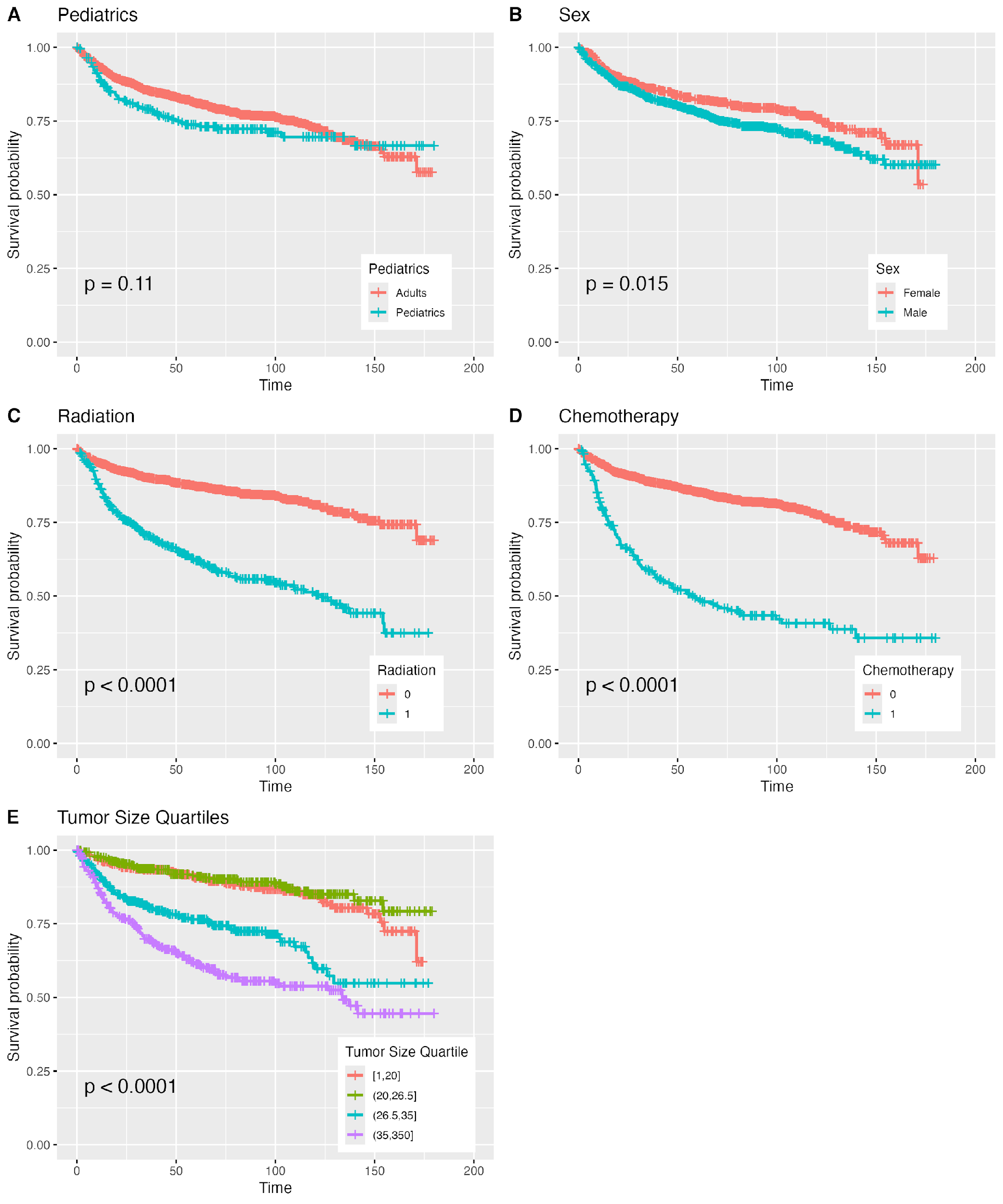


RMST = 101

RMST = 95

RMST = 99

RMST = 92

RMST = 106

RMST = 79

RMST = 104

RMST = 66

RMST = 107

RMST = 106

RMST = 89

RMST = 74

Supplemental 3: Kaplan Meier Curves for Surgery Cohort for Various Patient and Treatment Characteristics Stratified by GTR vs STR


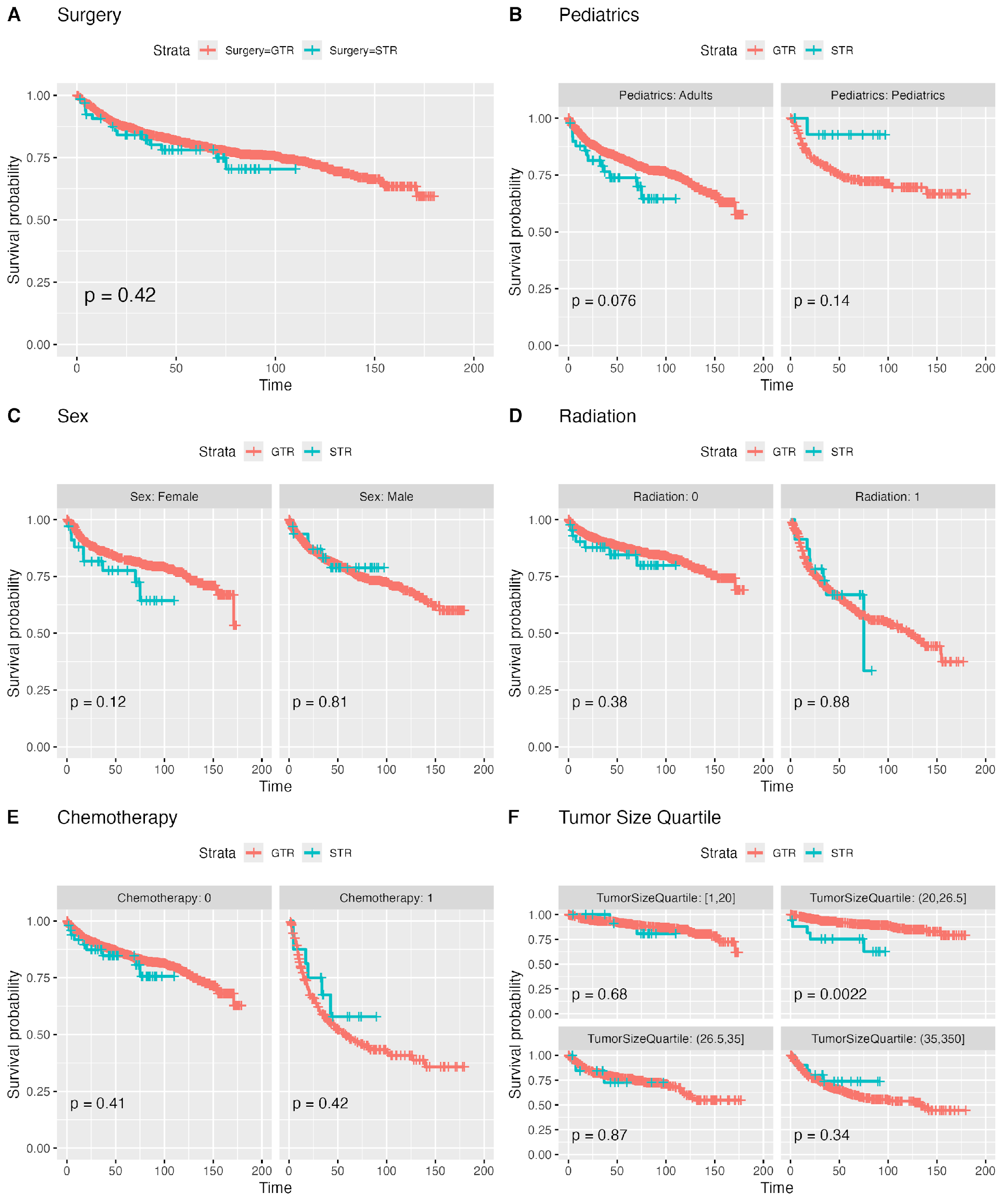


Supplemental 4: Machine Learning on Mortality using Random Forest Survival for Radiation Cohort and Summarized with SHAP Analysis and Feature Importance


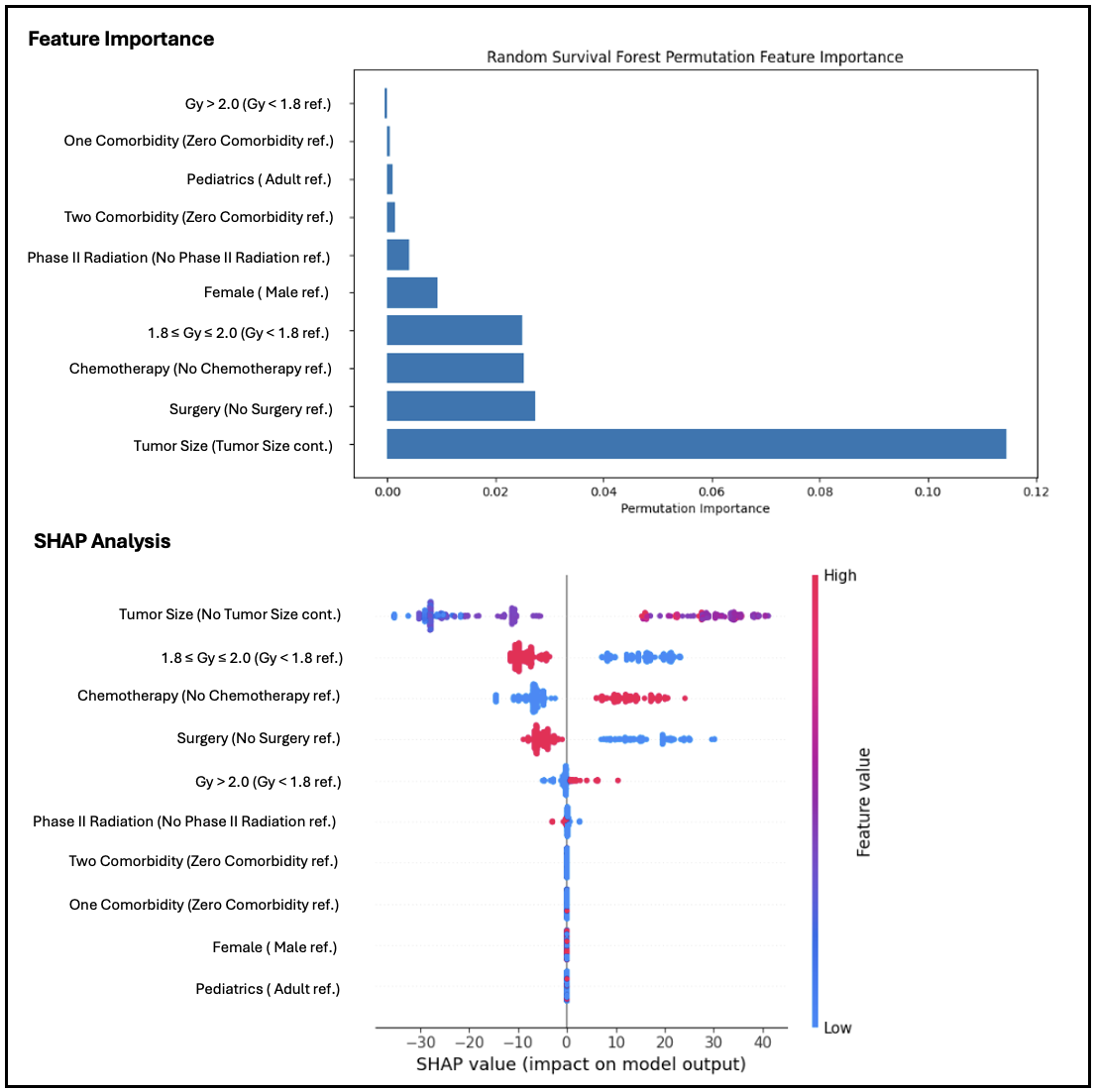


Supplemental 5: Kaplan Meier Curves for Whole Cohort for Various Patient and Treatment Characteristics Stratified by Radiation vs No Radiation


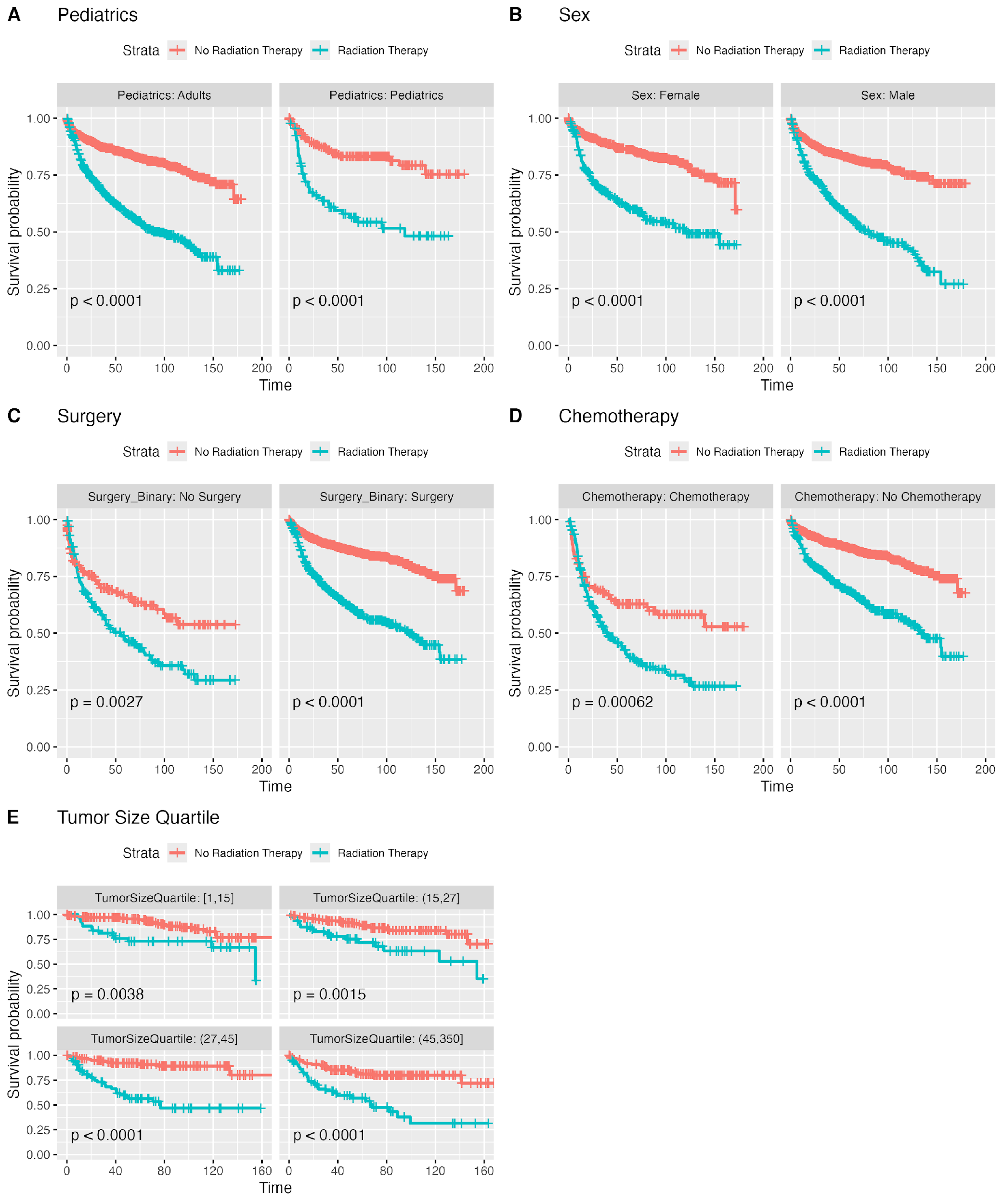


Supplemental 6: Kaplan Meier Curves for Whole Cohort for Various Patient and Treatment Characteristics Stratified by Chemotherapy vs No Chemotherapy


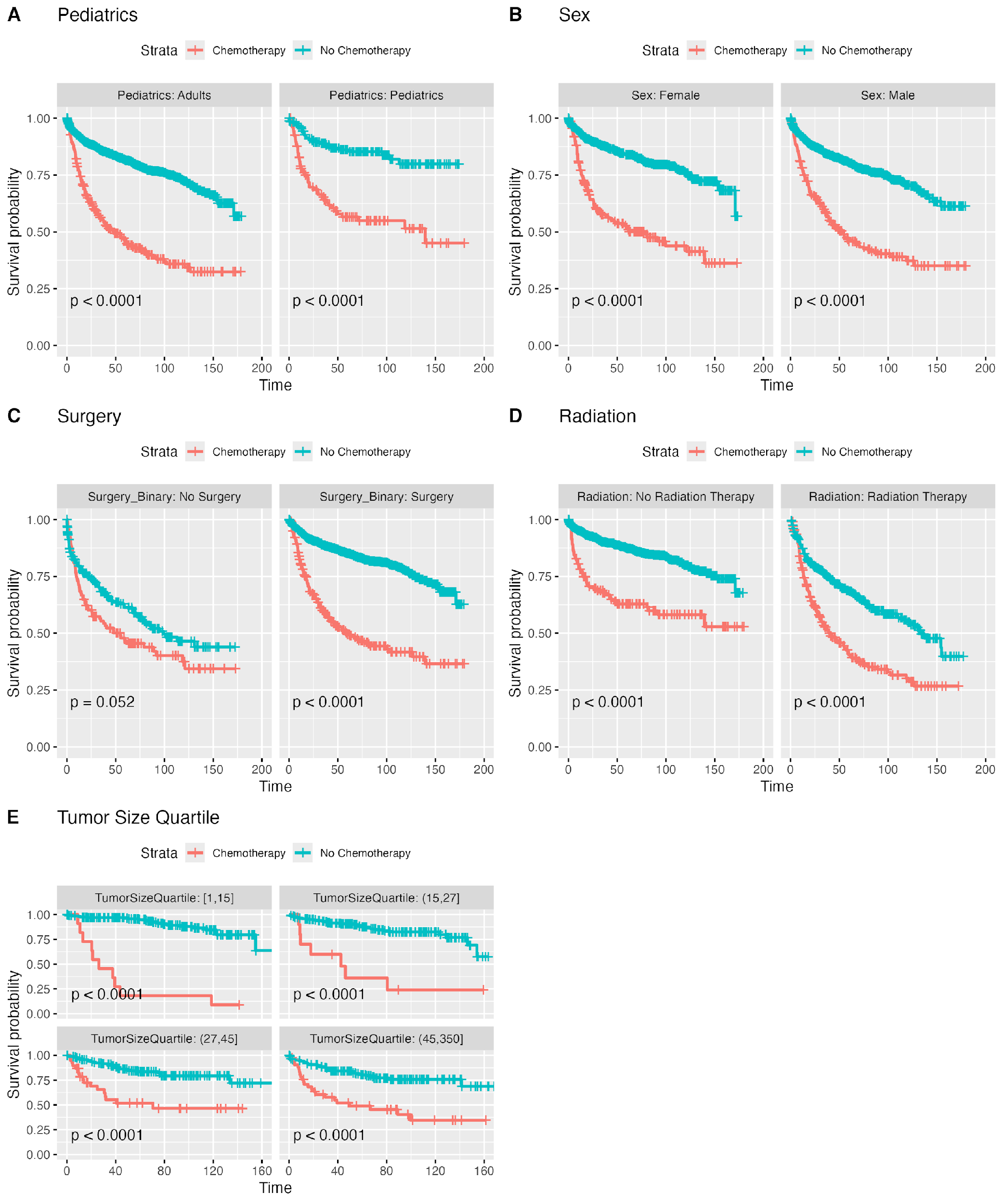

Supplement: Supplementary file 1 — Supplementary Material 1 [file 41598_2025_23070_MOESM1_ESM.docx]
